# Identification of New Transcription Factors that Can Promote Pluripotent Reprogramming

**DOI:** 10.1007/s12015-021-10220-z

**Published:** 2021-08-26

**Authors:** Ping Huang, Jieying Zhu, Yu Liu, Guihuan Liu, Ran Zhang, Dongwei Li, Duanqing Pei, Ping Zhu

**Affiliations:** 1 The Second School of Clinical Medicine, Southern Medical University, Guangzhou, 510515 Guangdong China; 2 Department of Cardiac Surgery, Guangdong Cardiovascular Institute, Guangdong Provincial People’s Hospital, Guangdong Academy of Medical Sciences, Guangzhou, 510100 Guangdong China; 3 CAS Key Laboratory of Regenerative Biology, South China Institute for Stem Cell Biology and Regenerative Medicine, Guangzhou Institutes of Biomedicine and Health, Chinese Academy of Sciences, Guangzhou, 510530 China; 4 Guangdong Provincial Key Laboratory of Stem Cell and Regenerative Medicine, South China Institute for Stem Cell Biology and Regenerative Medicine, Guangzhou Institutes of Biomedicine and Health, Chinese Academy of Sciences, Guangzhou, 510530 China; 5 Guangzhou Regenerative Medicine and Health Guangdong Laboratory, Guangzhou, 510005 China; 6 Guangdong Cardiovascular Institute, Guangdong Provincial People’s Hospital, Guangdong Academy of Medical Sciences, School of Medicine, South China University of Technology, Guangzhou, 510080 China; 7 Laboratory of Cell Fate Control, School of Life Sciences, Westlake University, Hangzhou, 310024 China; 8 Centre for Regenerative Medicine and Health, Hong Kong Institute of Science & Innovation, Chinese Academy of Sciences, Hong Kong, SAR People’s Republic of China

**Keywords:** TEAD2, TEAD4, ZIC3, Urine cells, Transcription factor, Induced pluripotent stem cell, Reprogramming, Myocardial differentiation

## Abstract

**Background:**

Four transcription factors, Oct4, Sox2, Klf4, and c-Myc (the Yamanka factors), can reprogram somatic cells to induced pluripotent stem cells (iPSCs). Many studies have provided a number of alternative combinations to the non-Yamanaka factors. However, it is clear that many additional transcription factors that can generate iPSCs remain to be discovered.

**Methods:**

The chromatin accessibility and transcriptional level of human embryonic stem cells and human urine cells were compared by Assay for Transposase-Accessible Chromatin with high-throughput sequencing (ATAC-seq) and RNA sequencing (RNA-seq) to identify potential reprogramming factors. Selected transcription factors were employed to reprogram urine cells, and the reprogramming efficiency was measured. Urine-derived iPSCs were detected for pluripotency by Immunofluorescence, quantitative polymerase chain reaction, RNA sequencing and teratoma formation test. Finally, we assessed the differentiation potential of the new iPSCs to cardiomyocytes in vitro.

**Results:**

ATAC-seq and RNA-seq datasets predicted TEAD2, TEAD4 and ZIC3 as potential factors involved in urine cell reprogramming. Transfection of TEAD2, TEAD4 and ZIC3 (in the presence of Yamanaka factors) significantly improved the reprogramming efficiency of urine cells. We confirmed that the newly generated iPSCs possessed pluripotency characteristics similar to normal H1 embryonic stem cells. We also confirmed that the new iPSCs could differentiate to functional cardiomyocytes.

**Conclusions:**

In conclusion, TEAD2, TEAD4 and ZIC3 can increase the efficiency of reprogramming human urine cells into iPSCs, and provides a new stem cell sources for the clinical application and modeling of cardiovascular disease.

**Graphical abstract:**

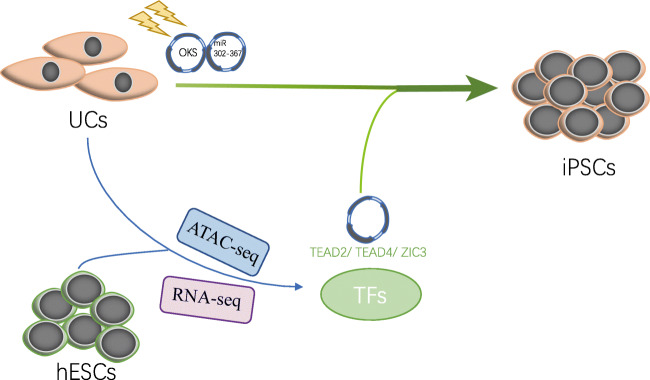

**Supplementary Information:**

The online version contains supplementary material available at 10.1007/s12015-021-10220-z.

## Background

The induced pluripotent stem cell (iPSC) technology pioneered by Yamanaka et al. [1] was one of the most exciting discoveries in biotechnology this century. This technology has great potential in precision medicine, disease modeling, and drug discovery, and it also avoids ethical issues related to the generation of human embryonic stem cells (hESCs) from human embryos. Moreover, the process of somatic cell reprogramming is a unique system for studying cell fate determination, which can help us to understand basic life processes such as embryonic development, senescence, and pathological processes.

The classic Yamanaka factors used to reprogram include the four transcription factors (TFs): OCT4, SOX2, KLF4, and c-MYC (OSKM) [1, 2]. It was understood relatively early that other TFs are capable of reprogramming and many research groups have provided several alternative combinations of non-Yamanaka factors that can reprogram. For instance, Yu et al. [3] used the OCT4, SOX2, NANOG and LIN28 systems to generate human iPSCs, and Wang et al. [4] demonstrated the combination of Jdp2, Jhdm1b, Mkk6, Glis1, Nanog, Essrb, and Sall4 (7F) for mouse iPSCs reprogramming.

OSKM appears to induce the expression of genes necessary for acquiring a pluripotent state [5, 6]. What’s more, our previous studies of chromatin accessibility showed that the transition from somatic cells to iPSCs was related to the opening and closing of specific chromatin sites [7]. Intriguingly, the chromatin data revealed that OSKM were not the only TFs that became active during reprogramming, and a plethora of different TF-families are potentially involved in reprogramming. This suggested additional TFs are capable of iPSC reprogramming.

Many types of human somatic cells can be reprogrammed into iPSCs [8–10]. Among them, urine cells (UCs) are a very promising source cell. UCs have obvious advantages: simplicity, low cost, non-invasiveness [11, 12]. Most importantly, human urine cells are readily available, compared to more common sources of somatic cells such as skin fibroblasts, or blood cells, and can be easily reprogrammed [13].

In this study, we used Assay for Transposase Accessible Chromatin with high-throughput sequencing (ATAC-seq) and RNA sequencing (RNA-seq) to analyze the chromatin accessibility and the transcriptome of human UCs and human embryonic stem cells (hESCs, H1). We found that the chromatin accessibility of the two kind cells were dynamic and interestingly, we found TEAD2, TEAD4, and ZIC3 DNA-binding motifs were enriched in pluripotent related open loci and highly expressed in hESCs, suggest those factors have a potential role in regulating the establishment and maintenance of the pluripotent network [4, 7]. Therefore, we verified their effect on UCs reprogramming and found that they can significantly improve human iPSCs reprogramming when transfected in combination with OSKM. The resulting reprogrammed cells were similar to hESCs as measured by several assays, including: morphology, immunofluorescence, qRT-PCR, teratoma formation. Finally, we show that the resulting iPSCs maintain normal differentiation capabilities and are capable of differentiation to cardiomyocytes.

## Methods

### Ethical Statement

The individuals in this study have signed written informed consent for donating UCs for stem cell generation. The experiments involving human subjects had been reviewed and approved by the Institutional Review Board at Guangdong Provincial People’s Hospital. The studies using mice have been approved by the Ethical Committee on Animal Experiments at Guangzhou Institutes of Biomedicine and Health, Chinese Academy of Sciences.

### Collection and Expansion of UCs

Two donors were recruited for the collection of urine cells (UCs) with informed consent based on IRB approval of Guangdong Provincial People’s Hospital. The purposes and procedures for isolating UCs and generating induced pluripotent stem cells were explained to donors in detail and questions, if any, were answered in full. We then obtained a formally signed consent form and collected urine from each donor. UCs were collected as described previously [14]. In brief, urine samples were collected at the mid-stream from 2 individuals. Each urine sample was centrifuged at 400 g for 10 min to collect the exfoliated cells. The primary UCs were then processed and cultured and passaged in medium consisting of REBM (renal epithelial basal medium) containing Single Quot Kit CC-4127 REGM (CC-3190, Lonza) / Dulbecco’s modified Eagle’s medium (DMEM)/high glucose (1×) (Hyclone) containing 100× MEM NEAA (Gibco), 100× glutaMAX-1 (Gibco), and 10% Fetal bovine serum (FBS) (Hyclone) 1:1 (the combination of them is referred to as RM). Cells were resuspended with RM containing 1× P/S and 1 μg/ml Promicin (ant-pm-2, Invivogen), and were then transferred to 6-well tissue culture dishes coated with 1% gelatin solution (Gibco). On the third day, 1 ml of primary culture medium was added. Starting on the fourth day, most of the medium was aspirated and 2.0 ml of RM was added. Then, this procedure was repeated every other day until the urine-derived cells were robustly expanded.

### Episomal Vectors Cloning

Human cDNAs encoding TEAD2, TEAD4, and ZIC3 were amplified by Polymerase Chain Reaction (PCR). Episomal vectors-pCEP4 was digested with NotI and BamHI and then those cDNAs were inserted into pCEP4 after enzyme digestion. The primers of cloning, PCR analysis, and qPCR were used according to a previous report [11]. Primers used in this research can be found in Table. S1.

### iPSC Generation

UCs (5 × 10^5^ to 1 × 10^6^) were individualized by 0.05% trypsin treatment and electroporated with indicated episomal plasmids using an Amaxa Basic Nucleofector Kit for primary mammalian epithelial cells, program T-020 (Lonza). The electroporated UCs were seeded onto matrigel (354,277, Becton Dickinson) pre-coated P6 wells. For the traditional reprogramming, cells that were electroporated with 3 μg of pEP4EO2SET2K (contains OCT4, SOX2, SV40LT, and KLF4) [12], and 2 μg of pCEP4-miR-302-367 cluster (contains miR-302b, c, a, d, and miR-367) [15]. The reprogramming medium was RM at days 0–1, and added five more small molecules A-83-01, Chir, Tzv, CPFT-a and NaB (5i) in RM medium at the early stage (days 2–9). Next, one more compound PD was added in mTeSR1 medium at the later stage (days 10–17) to maintain the reprogrammed iPSCs. When test the selected 3 candidate genes, 1.8 μg of pEP4EO2SET2K and 1.2 μg of pCEP4-miR-302-367 cluster plus 2 μg of pCEP4-TEAD2/pCEP4-TEAD4/pCEP4-ZIC3 were used and 2 μg of pCEP4-GFP served as positive controls. The reprogramming medium changed daily. The concentration of small molecules was shown as follows: 0.5 μM A-83-01 (SML0788-5MG, Sigma), 3 μM CHIR99021 (252917–06-9, Guangzhou Laura Biotech), 0.5 μM Tzv (1226056–71-8, Guangzhou Laura Biotech), 250 μM sodium butyrate (303410-100G, Sigma), 0.3 μM cyclic pifithrin-a (04–0040, Stemgent), and 0.5 μM PD0325901 (Guangzhou Laura Biotech). Each medium was changed every other day [16]. The iPSC colonies were picked at around days 15–18 and cultured in mTeSR1 on matrigel. The culture medium was changed daily. The iPSCs were passaged with Accutase (AT104–500, Innovative Cell Technologies).

### Alkaline Phosphatase (AP) Staining

AP staining was performed as described previously [17].

### PCR Analyses

PCR analysis of exogenous reprogramming factors and episomal backbone integration were performed as described previously [11]. Primers used in this research can be found in Table S1.

### qPCR Analyses

qPCR reactions were set up in technical triplicate with the ChamQ SYBR® qPCR Master Mix (Q711–02, Vazyme), all qPCR primers used in this manuscript can be found in Table S1.

### Immunofluorescence

Briefly, cells were fixed in 4% paraformaldehyde for 30 min at room temperature and blocked for 20 min at room temperature with 5% fetal bovine serum in PBS containing 0.1% Triton-X100. Then, the cells were incubated with primary antibody and appropriate second antibodies (Santa Cruz Biotechnology) diluted in 1% fetal bovine serum in PBS. Nuclei were stained with Gold Anti-fade Reagent with DAPI (Invitrogen). Then, the coverslips were mounted on the slides for observation on the confocal microscope (LSM800, Zeiss). The following primary antibodies were used in this project: OCT-4, SOX2, SSEA4, TRA-1-60(ab109884, Abcam), Cardiac Troponin T 647 (565,744, BD), and α-Actinin (Sarcomeric) monoclonal antibody (A7811, Sigma).

### Teratoma Assay

Teratoma assay was performed to examine the in vivo differentiation potential of human iPSCs. Immunodeficient NOD/SCID/IL2rg^−/−^ (NSI) mice lack mature T cells, B cells, and natural killer cells and therefore permit the engraftment of a wide range of primary human cells. iPSCs were collected with Accutase treatment, then resuspended by Matrigel and injected subcutaneously into 6-week-old immunocompromised NSI mice. Two injection groups were performed for each iPSC clone. After 4 weeks, teratomas were obtained from all injection groups. The teratomas were harvested and fixed in 4% paraformaldehyde (Jingxin Biotechnology). Samples were embedded in paraffin and processed with H&E staining in the Experimental Pathology Department of GIBH.

### Cell Culture

Human ES cell (H1) line was purchased from WiCell (WA01/H1; WiCell, USA). The integration-free human iPS cell line derived from urine cells of a healthy donor. Human ESCs and human UiPSCs were maintained on matrigel-coated plates (354,234, Corning) in mTeSR1 medium (85,850, stemcell). When the cell density reached to 50–60%, Accutase was used for digestion at 37 °C for 5 min. A small number of cells in mTeSR1 supplemented with 5 μM ROCK inhibitor Y-27632 (S1049, Selleckchem) for 24 h, and then changed to mTeSR1 medium without Y-27632.

### Cardiac Differentiation from hESCs and UiPSCs

Cardiac differentiation via GiWi2 protocol using RPMI differentiation medium was performed as described previously [18]. hPSCs including hESCs and UiPSCs were dissociated into single cells with Accutase at 37 °C for 5 min and then seeded onto a matrigel-coated cell culture dish at a density of 100,000–200,000 cells/cm^2^ in mTeSR1 supplemented with 5 μM ROCK inhibitor Y-27632 (day −2) for 24 h. Cells were then cultured in mTeSR1 and changed medium daily. On day 0, cells were treated with 6 μM CHIR99021 **(**CT99021, Selleckchem) in RPMI medium for 24 h (day 0 to day 1). On day 3, half of the medium was changed with a fresh RPMI medium containing 2.5 μM IWP2 (3533, Tocris). On day 5, the entire medium was changed with the RPMI medium. Cells were maintained in cardiomyocyte maintenance medium (CMM: RPMI supplemented with ITS-A Supplement (100X) (51,300,044, Gibco) and 200 μg/ml L-Ascorbic acid 2-phosphate sesquimagnesium salt hydrate (A8960, Sigma)) starting from day 7, with the medium changed every 2 days.

### Flow Cytometry Analysis

To analyze the efficiency of myocardial cell differentiation, about 1 × 10^6^ cells on day 15 of differentiation from hESCs and UiPSCs were harvested by using standard techniques. Briefly, cells on day 15 of differentiation were trypsinized and fixed with 4% paraformaldehyde for 20 min at room temperature. The cells were then washed with FACS buffer (PBS containing 2% FBS) and resuspended in 0.5% TritonX-100 for permeabilization by incubating for 10 min at room temperature. After washing, cells were sequentially incubated with fluorescently labeled antibody (Alexa Fluor 647-conjugated mouse anti-Cardiac Troponin T) overnight. Flow cytometry data were acquired by using BD Accuri C6 Flow analyzer and were analyzed using FlowJo vX.0.7 software.

### ATAC-Seq

ATAC-seq was performed as previously described [7, 19, 20]. In brief, a total of 50,000 cells were washed once with 50 μL of cold PBS and resuspended in 50 μL lysis buffer (10 mM Tris-HCl pH 7.4, 10 mM NaCl, 3 mM MgCl_2_, 0.2% (*v*/v) IGEPAL CA-630). The suspension of nuclei was then centrifuged for 10 min at 500 g at 4 °C, followed by the addition of 50 μL transposition reaction mix (25 μL TD buffer, 2.5 μL Tn5 transposase, and 22.5 μL nuclease-free H_2_O) of Nextera DNA Library Preparation Kit (96 samples) (FC-121-1031, Illumina). Samples were then PCR amplified and incubated at 37 °C for 30 min. DNA was isolated using a MinElute Kit (QIAGEN). ATAC-seq libraries were first subjected to 5 cycles of pre-amplification. To determine the suitable number of cycles required for the second round of PCR the library was assessed by quantitative PCR as described [20], and the library was then PCR amplified for the appropriate number of cycles. Libraries were purified with a Qiaquick PCR (QIAGEN) column. Library concentration was measured by using a KAPA Library Quantification kit (KK4824) according to the manufacturer’s instructions. Library integrity was checked by gel electrophoresis. Finally, the ATAC library was sequenced on a NextSeq 500 using a NextSeq 500 High Output Kit v2 (150 cycles) (FC-404-2002, Illumina) according to the manufacturer’s instruction.

### RNA-Seq and Gene Expression Analysis

Total mRNA was isolated from hUCs, iPSCs, and ESCs. RNA-seq libraries were constructed using the VAHTSTM mRNA-seq V3 Library Prep Kit for Illumina® (Vazyme). RNA samples were sequenced by Annoroad Gene Technology Corporation. The clustering of the index-coded samples was performed on a cBot cluster generation system using HiSeq PE Cluster Kit v4-cBot-HS (Illumina) according to the manufacturer’s instructions. After cluster generation, the libraries were sequenced on an Illumina platform. RNA-seq was processed as described previously [21], briefly reads were using aligned to a transcriptome index generated from the Ensembl annotations (v79), using RSEM [22], bowtie2 [23], and normalized using EDASeq [24]. RNA-seq data were expressed in units of GC-normalized tag counts.

### ATAC-Seq Bioinformatic Analysis and Peak Calling

All sequencing data were mapped onto the hg38 human genome assembly using bowtie2 (−very-sensitive). Low quality mapped reads were removed using samtools (view –q 35) and unique reads mapping to a single genomic location and strand were kept. We removed mitochondrial sequences using ‘grep –v ‘chrM’. Biological replicates were merged, and peaks were called using Dfilter (Kumar et al., 2013) (with the settings: -bs = 100 –ks = 60 –refine). BigWig files were produced using genomeCoverageBed from Bedtools (scale = 107/ < each_sample’s_total_unique_reads >) and then bedGraphToBigWig. Gene ontology and gene expression measures were called by first collecting all TSSs within 10 kb of an ATAC-seq peak, and then performing GO analysis with GOseq [25], or measuring gene expression. Other analysis was performed using deepTools [26], HOMER [27], or glbase [28].

### Statistical Analysis

The data are expressed as the mean ± SEM of at least three independent experiments. For comparison of different groups where appropriate, T-test were used for determining statistical significance (*, *p* ≤ 0.05; **, *p* ≤ 0.01; and ***, *p* ≤ 0.001 were considered significant).

## Results

### Chromatin Accessibility Dynamics Reveal Key Transcription Factors in Pluripotent Stem Cells

**Fig. 1:**
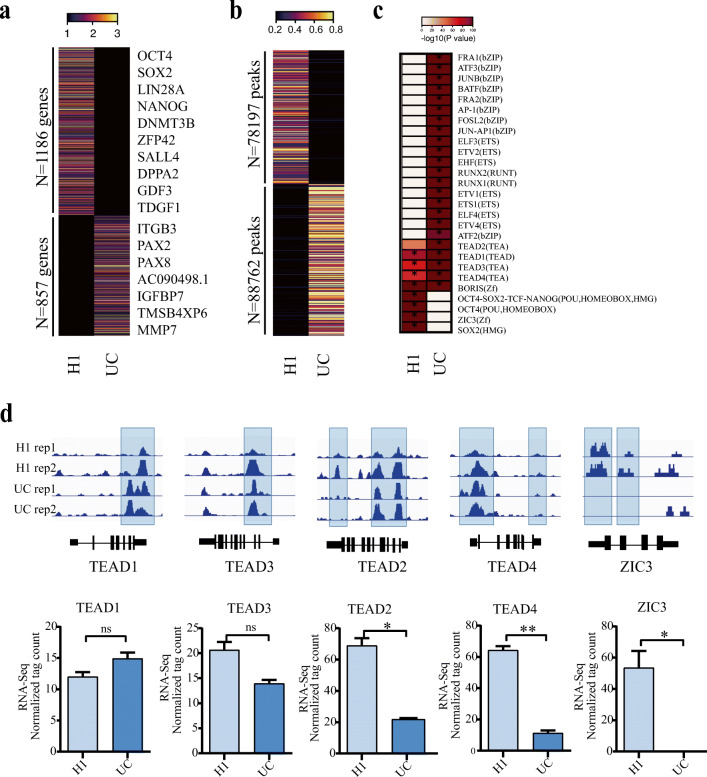
Screen candidate reprogramming factors by analysing chromatin accessibility and transcriptome data. **a** Heatmap of RNA-seq analysis results of UCs and hESCs (H1). There are 1186 highly expressed genes in hESCs (H1) and 857 highly expressed genes in UCs. **b** ATAC-seq chromatin status. In order to make the data clearer, the sequencing results of the two replicates of UCs and hESCs (H1) were merged into one dataset using the mean value. There are 88,762 peaks opened in UCs but closed in hESCs (H1) and 78,197 peaks closed in UCs but opened in hESCs (H1). **c** DNA motif enrichment analysis of two groups of ATAC-seq data by HOMER [27]. **d** Chromatin landscape and gene expression value of the target genes (UC rep1 and UC rep2 represent ATAC-seq data from two donors). RNA-seq data are presented as mean ± SEM, *n* = 2. ns P>0.05, *P < 0.05, **P < 0.01, unpaired two tailed student-t-test

To screen for new TFs that could enhance human iPSCs reprogramming, we collected RNA-seq and ATAC-seq data from UCs (Fig. S1) from healthy donors, and human embryonic stem cell line (H1) (Fig. S2). All the UCs’ sequencing data were from two donors, we merged the two repeats and present the mean value of the ATAC-seq and RNA-seq data. Through the analysis of the transcriptome profile, 1186 and 857 highly expressed genes were discovered in the H1 and UCs respectively (Fig. 1a). By comparing the open peaks between H1 and UCs, the dataset reveals two basic groups, 78,197 peaks open in H1 but closed in UCs and 88,762 peaks open in UCs but closed in H1 (Fig. 1b). In the open peaks of H1, we detected the DNA binding motifs for several known pluripotent transcription factors (TFs) such as OCT4, SOX2, and the composite OCT4-SOX2 motif (OCT4-SOX2-NANOG). We also detected binding motifs for ZIC3, which has previously been demonstrated as a key regulator in maintain mouse embryonic stem cells (mESCs), and could enhance generate mouse induced pluripotent stem cells (miPSCs) [29, 30]. Additionally, we observed enrichment for motifs in the open peaks for the TEAD (TEA domain) family of TFs, TEAD1, TEAD2, TEAD3, and TEAD4. These motifs were enriched in both the open peaks of UCs and H1s (Fig. 1c and d). When then looked at the gene expression data, TEAD2, TEAD4 and ZIC3 were higher expressed in H1, however, the expression of TEAD1 and TEAD3 in UCs were similar with H1 (Fig. 1d). Overall, these analyses suggest TEAD2, TEAD4 and ZIC3 are involved in maintaining pluripotency, and may have the potential for promote human iPSCs reprogramming.

### TEAD2, TEAD4 and ZIC3 Enhance Reprogram UCs into iPSCs

**Fig. 2:**
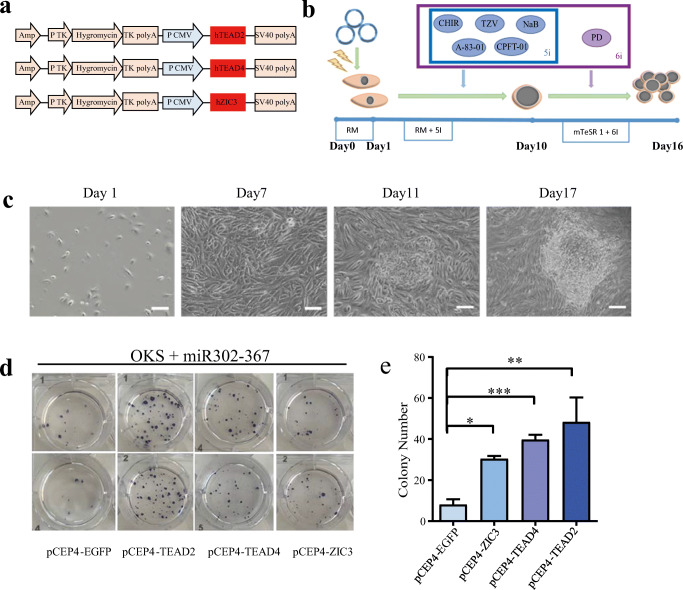
TEAD2, TEAD4 and ZIC3 can promote reprogramming of human UCs into iPSCs. **a** Construction map of Episomal vectors of TEAD2, TEAD4 and ZIC3: pCEP4-TEAD2, pCEP4-TEAD4, pCEP4-ZIC3. **b** Reprogramming protocol: add 5i to RM medium during induction period, and add 5i + PD to mTeSR1 medium during proliferation period. **c** The morphological changes of induced cells in different time points during the reprogramming process. **d** AP staining of each group iPSCs induced from UCs. **e** The number of AP+ clone number of each group of UCs. Data represented as mean ± SEM from three independent assays. *P < 0.05, **P < 0.01, ***P < 0.001, unpaired two tailed student-t-test

To investigate the role of TEAD2, TEAD4 and ZIC3 in reprogramming, we constructed three overexpression vectors pCEP4-TEAD2, pCEP4-TEAD4 and pCEP4-ZIC3 (Fig. 2a). We first confirmed that the overexpression vectors could induce expression of the target genes TEAD2, TEAD4, ZIC3 in HEK-293 T (Fig. S3). Following the schematic showing the reprogram protocol of UCs into iPSCs with non-integrated episomal vectors [16] (Fig. 2b), we delivered the reprogramming vectors by electroporation. The morphology of UCs rapidly changed and the cells began to aggregate, and formed clusters at day 7. After changing the reprogramming medium at day 10, the cells continued to reprogram and formed obvious cell clones at day 16, with clear boundaries and slight bulges (Fig. 2c). On day 16, we performed AP staining and counted the number of AP+ clones (Fig. 2d). The number of colonies showed that TEAD2, TEAD4, and ZIC3 co-transfected with OSK + miR302–367 could significantly increase reprogramming of UCs to UiPSCs (Fig. 2e). This suggests the TFs enriched in pluripotent-specific open peaks can enhance reprogramming.

### The Quality of UiPSCs Generated by TEAD2, TEAD4 and ZIC3

**Fig. 3:**
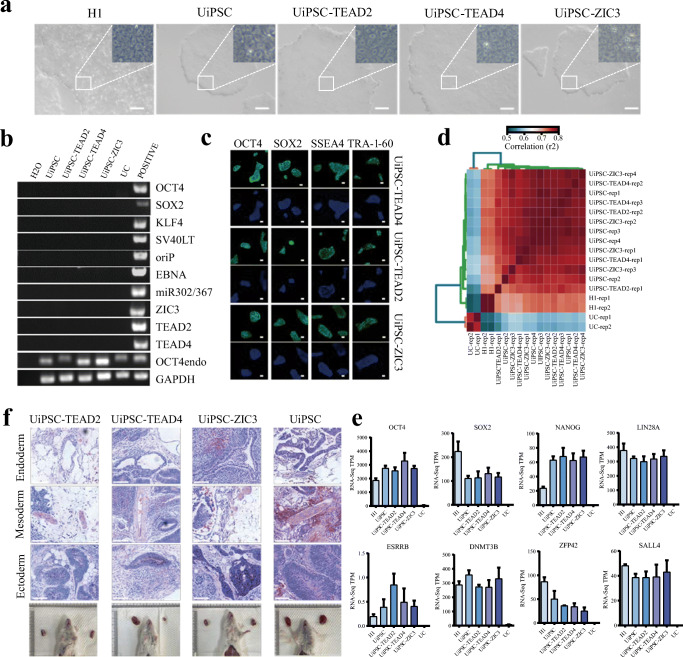
Characteristics of the reprogrammed UiPSCs-TEAD2, UiPSCs-TEAD4 and UiPSCs-ZIC3 cell lines. **a** The morphology of UiPSCs-TEAD2, UiPSCs-TEAD4 and UiPSCs-ZIC3 clones are similar to H1 and UiPSCs (bar: 200 μm). **b** Detection of exogenous reprogramming factor integration by PCR analysis using genomic DNA in the UiPSCs clones. **c** immunofluorescence images of the pluripotency markers (OCT4, SOX2 SSEA4 and TRA-1-60) in UiPSCs-TEAD2, UiPSCs-TEAD4 and UiPSCs-ZIC3 cells (bar: 50 μm). **d** The R^2^ correlation coefficient matrix of all versus all RNA-seq datasets as indicated. **e** The expression level for select pluripotent genes from RNA-seq data. Data represented as mean ± SEM from two replicates. **f** Teratoma assay of UiPSCs-TEAD2, UiPSCs-TEAD4, UiPSCs-ZIC3 and control UiPSCs. H&E staining was performed after 4 weeks subcutaneous injection, each image presents histology consistent with endoderm, mesoderm or ectoderm. (bar: 90 μm)

The UiPSCs induced by OSK and miR-302-367 clusters was named UiPSCs. And the iPSCs generated with the addition of the TFs, TEAD2, TEAD4 and ZIC3, were named as UiPSCs-TEAD2, UiPSCs-TEAD4 and UiPSCs-ZIC3, respectively. To analyze the quality of UiPSCs, we performed AP staining, qRT-PCR, and immunofluorescence (Fig. S4a-c). The expression of pluripotent genes such as *SOX2*, *OCT4 (POU5F1)*, *NANOG*, *LIN28A*, *SALL4* and *GDF3* of UiPSCs were similar to hESCs (Fig. S4b). To identify the pluripotency of UiPSCs-TEAD2, UiPSCs-TEAD4, and UiPSCs-ZIC3, we picked up and passaged the ESC-like colonies for at least 10 passages. When compared with H1 and UiPSCs, the colonies exhibited similar cellular morphology (Fig. 3a). PCR did not detect any integration of the episomal exogenous reprogramming factors (Fig. 3b). Immunofluorescence was used to detect pluripotent markers: OCT4, SSEA4, TRA-1-60, and NANOG, and they were all present in the UiPSCs-TEAD2, UiPSCs-TEAD4 and UiPSCs-ZIC3 clones (Fig. 3c). To analyze the transcriptome of UiPSCs, we performed RNA-seq on UiPSCs-TEAD2, UiPSCs-TEAD4, UiPSCs-ZIC3, UiPSCs, H1 and UCs. Cross-correlation showed good correlation between the UiPSCs-TEAD2, UiPSCs-TEAD4, and UiPSCs-ZIC3, indicating similar overall expression profiles to UiPSCs and H1, but quite distinct from UCs (Fig. 3d). According to the RNA-seq data, we further compared the expression of 8 pluripotent factors, such as *OCT4* (POU5F1), *SOX2*, *NANOG*, *LIN28A*, *ESRRB*, *DNMT3B*, *ZFP42*, *SALL4.* The expression of these 8 genes in UiPSCs-TEAD2, UiPSCs-TEAD4, and UiPSCs-ZIC3 were comparable to H1 and UiPSCs (Fig. 3e). Furthermore, we examined the differentiation potential of these UiPSCs in vivo. By injection of UiPSCs-TEAD2, UiPSCs-TEAD4, UiPSCs-ZIC3 and UiPSCs under the skin of NSI mice and all tests yielded tumors after one month. HE staining showed all the teratoma contain tissues of the three typical germ layers (Fig. 3f). These results indicated that UiPSCs-TEAD2, UiPSCs-TEAD4, and UiPSCs-ZIC3 possessed pluripotency similar to hESCs and UiPSCs.

### UiPSCs Can Differentiate into Myocardial Cells

**Fig. 4:**
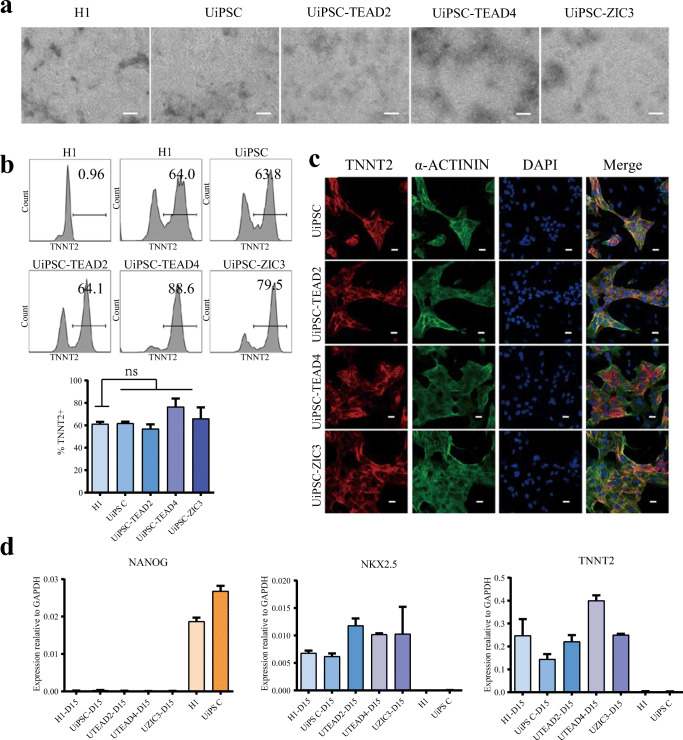
Induce UiPSCs-TEAD2, UiPSCs-TEAD4, and UiPSCs-ZIC3 differentiate to myocardial cells. **a** The cell morphology of induced myocardial cells from H1, UiPSCs, UiPSCs-TEAD2, UiPSCs-TEAD4 and UiPSCs-ZIC3 (bar: 200 μm). **b** FACS assay to detect the proportion of TNNT2+ cells after 15 days myocardial cells differentiation. **c** Immunofluorescence experiment to detect the expression of myocardial markers TNNT2 and α-actinin in differentiated myocardial cells. The nuclei were stained with DAPI (bar: 20 μm). **d** qPCR was used to detect the expression of pluripotency markers (*NANOG*) and myocardial markers (*NKX2.5* and *TNNT2*) in differentiated cells and pluripotent cells. Data represented as mean ± SEM from three independent assays. *P < 0.05, **P < 0.01, ***P < 0.001, unpaired two tailed student-t-test

To explore the differentiation potential of UiPSCs-TEAD2, UiPSCs-TEAD4 and UiPSCs-ZIC3, we induced those UiPSCs to differentiate into myocardial cells in vitro using an albumin-free GiWi (named GiWi2) protocol reported by Lian et al. [19]. Among the 15 days of cardiac differentiation, the majority of cells exhibited spontaneous contractions, and typical cardiomyocyte morphology (Fig. 4a, video. S1, video. S2, video. S3, video. S4, video. S5). When compare the TNNT2+ cells by flow cytometry, UiPSCs-TEAD2, UiPSCs-TEAD4 and UiPSCs-ZIC3 could generated more than 60% TNNT2+ cells, similar to H1 and UiPSCs (Fig. 4b). Moreover, the immunofluorescence images show that all the cardiomyocyte induced from UiPSCs, UiPSCs-TEAD2, UiPSCs-TEAD4 and UiPSCs-ZIC3 expressed the myocardial cell markers TNNT2 and α-actinin (Fig. 4c). In addition, RT-qPCR revealed the upregulation of myocardial cell specific marker genes *NKX2.5* and *TNNT2* and downregulation of pluripotent gene *NANOG* in myocardial cells differentiated from UiPSCs-TEAD2, UiPSCs-TEAD4, and UiPSCs-ZIC3, as compared with undifferentiated H1 and UiPSCs (Fig. 4d). The results suggest UiPSCs obtained from TEAD2, TEAD4, and ZIC3 have the potential to differentiate into cardiomyocytes.

## Discussion

In 2006, Takahashi and Yamanaka. reported induced pluripotent stem cells (iPSCs) [1], Since then many improvements to have been enacted to improve the generation of iPSCs, however the mechanism of reprogramming is still unclear. In our previous study, we found that chromatin dynamics could regulate cell fate decisions [7]. Through the analysis of the transcriptome and chromatin accessibility during reprogramming we developed a new 7 factors (7F) reprogramming system, which could generate high quality iPSCs with only 4 days induction [4]. However, whilst mouse iPSC generation is now relatively efficient, human iPSCs reprogramming remains inefficient and difficult. Applying the same 7F logic to human, we found three new factors: TEAD2, TEAD4 and ZIC3 that could enhance the generation of human iPSCs.

ZIC3 is a member of the ZIC (zinc-finger of the cerebellum) gene family. It belongs to the GLI superfamily of zinc-finger transcription factors and is homologous to the Opa gene of Drosophila [31]. ZIC3 protein contains 1 DNA binding domain, 2 nuclear localization signals (NLS), and 1 nuclear export signal (NES). And the zinc finger domain (ZFD) of this protein contains 5 tandem zinc finger motifs. ZIC3 participates in a variety of physiological processes, and its mutations can cause a variety of developmental abnormalities, which is especially important for left-right symmetry [10]. ZIC3 also plays a role in maintaining the pluripotency of embryonic stem cells. Lim Ls et al. [29] confirmed that ZIC3 was the direct target gene of OCT4, NANOG, and SOX2. ZIC3 was continuously expressed in embryonic stem cells, and is down regulated when stem cells begin to differentiate. Lim Ls et al. [29] speculated that ZIC3 maintain the pluripotency of embryonic stem cells by inhibiting the differentiation of endoderm. Yang et al. [30] showed that ZIC3 was a potential regulator of cell fate transitions during embryonic stem cell differentiation. In our study we found ZIC3 also could promotes reprogramming of human UCs into iPSCs.

The Hippo signaling pathway was discovered in the past two decades, and plays an important role in regulating organ size and tissue homeostasis, embryo development, and tumor-related signal transduction pathways [32, 33]. The transcriptional co-activator YAP/TAZ are the core part of the Hippo pathway. It regulates the expression of downstream genes by combining with transcription factors such as TEAD. Nishioka et al. [34] found that TEAD4 was a key factor in the formation of trophectoderm, and its role was closely related to the Hippo signaling pathway. Studies found that the TEA domain of TEAD4 have a wide range of interactions with DNA double strands, including the main and auxiliary grooves of the DNA helix. Mutations at any site of TEAD4 can significantly reduce its occupancy in the promoter region of the target gene, thereby severely impairing YAP-induced TEAD4 transactivation and gene transcription [35].

TEAD2 is another member of the TEAD family but to date it has not been well studied. TEAD2, like other members of the TEAD family, can bind to YAP (an important member of the evolutionarily conserved Hippo pathway [36] and activate the expression of anti-apoptosis and proliferation genes. TEAD2 was considered a new tumor prognostic factor [37]. Tamm et al. [38] revealed that the Yes-YAP-TEAD2 signaling system was downstream of the LIF signaling pathway, which was of great significance to the self-renewal of mouse ESCs. It has been reported that TEAD4 can improve the reprogramming efficiency of human fibroblasts [39].

Urine-derived iPSCs can serve as a modeling tool to study rare human diseases. Previous studies have shown that Urine-derived iPSCs can further differentiate to a variety of cell types, including cardiomyocytes [40–44]. In this study, we show that all the UiPSCs generated by reprogramming cocktail(OSK + miR302–367)with additional TFs such as TEAD2, TEAD4 or ZIC3 could differentiated to functional cardiomyocytes. These results indicated that the expression of TEAD2, TEAD4 and ZIC3 during human iPSCs reprogramming did not affect its differentiation ability in vitro.

In summary, we showed that TEAD2, TEAD4 and ZIC3 can enhance the efficiency of reprogram human UCs into iPSCs by the traditional reprogram cocktail (OSK + miR302–367). Further study could use this more efficiency reprogramming system to reprogram patient UCs into iPSCs and establish disease modeling for studying disease mechanisms, screening patient-specific drug and developing potential therapies.

## Conclusions

We designed a strategy to screen new factors that could enhance human iPSC reprogramming and identified three potential transcription factors: TEAD2, TEAD4 and ZIC3. The results presented here not only confirmed the role of TEAD4 in promoting human iPSC reprogramming but also provided evidence of TEAD2 and ZIC3 involvement in human reprogramming systems. We finally conclude that these transcription factors did not affect the differentiation ability of UiPSCs in vitro and could be used for further clinical application of stem cells therapy in cardiovascular disease.

## Supplementary Information

Fig. S1 Morphology of urine cells obtained from volunteer donors (before induction) (bar: 200 μm). (PNG 1622 kb)

High resolution image (TIF 2346 kb)

Fig. S2 Per base sequence quality of FASTQC Report of ATAC-seq. UC rp1 and UC rp2 represent urine cell samples from two donors, respectively. H1 rp1 and H1 rp2 represent two ATAC-seq libraries of human embryo stem cells. (PNG 9361 kb)

High resolution image (TIF 3005 kb)

Fig. S3 After transiently transfecting pCEP4-TEAD2, pCEP4-TEAD4 and pCEP4-ZIC3 into HEM-293 T cells, the expression of TEAD2, TEAD4 and ZIC3 was significantly higher than control cells transfected with pCEP4-GFP. Data represented as mean ± SEM from three independent assays. *P < 0.05, **P < 0.01, ***P < 0.001. (PNG 1995 kb)

High resolution image (TIF 738 kb)

Fig. S4 The characteristics of UiPSCs. **a** AP staining shown AP positive clones after 17 days iPSCs reprogramming with OSK plus miR302–367 cluster (bar = 100 μm). **b** The expression level of pluripotent genes OCT4, SOX2, NANOG, LIN28A, SALL4, GDF3 in UiPSCs, H1 and UCs. Data represented as mean ± SEM from three independent assays. Data represented as mean ± SEM from three independent assays. *P < 0.05, **P < 0.01, ***P < 0.001. **c** Immunostaining of OCT4, SOX2, SSA4, TRA-1-60 in UiPSCs (bar: 100 μm). (PNG 5925 kb)

High resolution image (TIF 2291 kb)

Video. S1 The cardiomyocyte contraction movie about H1 induced cardiomyocyte differentiation at day 15. (MP4 4128 kb)

Video. S2 The cardiomyocyte contraction movie about UiPSCs induced cardiomyocyte differentiation at day 15. (MP4 4547 kb)

Video. S3 The cardiomyocyte contraction movie about UiPSCs-TEAD2 induced cardiomyocyte differentiation at day 15. (MP4 2264 kb)

Video. S4 The cardiomyocyte contraction movie about UiPSCs-TEAD4 induced cardiomyocyte differentiation at day 15. (MP4 3310 kb)

Video. S5 The cardiomyocyte contraction movie about UiPSCs-ZIC3 induced cardiomyocyte differentiation at day 15. (MP4 5655 kb)

Table S1 Primer list. (XLSX 12 kb)

## Data Availability

The datasets used and analyzed during the current study are available from the corresponding author on reasonable request. The ATAC-seq and RNA-seq data described in this study was deposited with the gene expression omnibus with the accession number GEO: GSE168392.

## Abbreviations

ATAC-seq: Assay for Transposase Accessible Chromatin with high-throughput sequencing
RNA-seq: RNA sequencing.
UCs: Urine cells; ESCs: Embryo stem cells
iPSCs: Induced pluripotent stem cells
OSKM: OCT4, SOX2, KLF4, and c-MYC
AP: Alkaline phosphatase
TFs: Transcription factors
CMs: Cardiomyocytes
PCR: Polymerase Chain Reaction
REBM: renal epithelial basal medium
DMEM: Dulbecco’s modified Eagle’s medium
FBS: Fetal bovine serum
IF: Immunofluorescence
KSR: Knockout serum replacement

## References

[1] Takahashi K, Yamanaka S (2006). Induction of pluripotent stem cells from mouse embryonic and adult fibroblast cultures by defined factors. Cell.

[2] Takahashi K, Tanabe K, Ohnuki M, Narita M, Ichisaka T, Tomoda K, Yamanaka S (2007). Induction of pluripotent stem cells from adult human fibroblasts by defined factors. Cell.

[3] Yu J, Vodyanik MA, Smuga-Otto K, Antosiewicz-Bourget J, Frane JL, Tian S, Nie J, Jonsdottir GA, Ruotti V, Stewart R, Slukvin II, Thomson JA (2007). Induced pluripotent stem cell lines derived from human somatic cells. Science.

[4] Wang B, Wu L, Li D, Liu Y, Guo J, Li C, Yao Y, Wang Y, Zhao G, Wang X, Fu M, Liu H, Cao S, Wu C, Yu S, Zhou C, Qin Y, Kuang J, Ming J, Chu S, Yang X, Zhu P, Pan G, Chen J, Liu J, Pei D (2019). Induction of pluripotent stem cells from mouse embryonic fibroblasts by Jdp2-Jhdm1b-Mkk6-Glis1-Nanog-Essrb-Sall4. Cell Reports.

[5] Huang J, Chen T, Liu X, Jiang J, Li J, Li D, Liu XS, Li W, Kang J, Pei G (2009). More synergetic cooperation of Yamanaka factors in induced pluripotent stem cells than in embryonic stem cells. Cell Research.

[6] Sridharan R, Tchieu J, Mason MJ, Yachechko R, Kuoy E, Horvath S, Zhou Q, Plath K (2009). Role of the murine reprogramming factors in the induction of pluripotency. Cell.

[7] Li D, Liu J, Yang X, Zhou C, Guo J, Wu C, Qin Y, Guo L, He J, Yu S, Liu H, Wang X, Wu F, Kuang J, Hutchins AP, Chen J, Pei D (2017). Chromatin accessibility dynamics during iPSC reprogramming. Cell Stem Cell.

[8] Aasen T, Raya A, Barrero MJ, Garreta E, Consiglio A, Gonzalez F, Vassena R, Bilic J, Pekarik V, Tiscornia G, Edel M, Boue S, Izpisua Belmonte JC (2008). Efficient and rapid generation of induced pluripotent stem cells from human keratinocytes. Nature Biotechnology.

[9] Cai J, Li W, Su H, Qin D, Yang J, Zhu F, Xu J, He W, Guo X, Labuda K, Peterbauer A, Wolbank S, Zhong M, Li Z, Wu W, So KF, Redl H, Zeng L, Esteban MA, Pei D (2010). Generation of human induced pluripotent stem cells from umbilical cord matrix and amniotic membrane mesenchymal cells. The Journal of Biological Chemistry.

[10] Seki T, Yuasa S, Oda M, Egashira T, Yae K, Kusumoto D, Nakata H, Tohyama S, Hashimoto H, Kodaira M, Okada Y, Seimiya H, Fusaki N, Hasegawa M, Fukuda K (2010). Generation of induced pluripotent stem cells from human terminally differentiated circulating T cells. Cell Stem Cell.

[11] Xue Y, Cai X, Wang L, Liao B, Zhang H, Shan Y, Chen Q, Zhou T, Li X, Hou J, Chen S, Luo R, Qin D, Pei D, Pan G (2013). Generating a non-integrating human induced pluripotent stem cell bank from urine-derived cells. PLoS One.

[12] Yu J, Hu K, Smuga-Otto K, Tian S, Stewart R, Slukvin II, Thomson JA (2009). Human induced pluripotent stem cells free of vector and transgene sequences. Science.

[13] Zhou T, Benda C, Duzinger S, Huang Y, Li X, Li Y, Guo X, Cao G, Chen S, Hao L, Chan YC, Ng KM, Ho JC, Wieser M, Wu J, Redl H, Tse HF, Grillari J, Grillari-Voglauer R, Pei D, Esteban MA (2011). Generation of induced pluripotent stem cells from urine. J Am Soc Nephrol.

[14] Zhou T, Benda C, Dunzinger S, Huang Y, Ho JC, Yang J, Wang Y, Zhang Y, Zhuang Q, Li Y, Bao X, Tse HF, Grillari J, Grillari-Voglauer R, Pei D, Esteban MA (2012). Generation of human induced pluripotent stem cells from urine samples. Nature Protocols.

[15] Liao B, Bao X, Liu L, Feng S, Zovoilis A, Liu W, Xue Y, Cai J, Guo X, Qin B, Zhang R, Wu J, Lai L, Teng M, Niu L, Zhang B, Esteban MA, Pei D (2011). MicroRNA cluster 302-367 enhances somatic cell reprogramming by accelerating a mesenchymal-to-epithelial transition. The Journal of Biological Chemistry.

[16] Qin D, Gan Y, Shao K, Wang H, Li W, Wang T, He W, Xu J, Zhang Y, Kou Z, Zeng L, Sheng G, Esteban MA, Gao S, Pei D (2008). Mouse meningiocytes express Sox2 and yield high efficiency of chimeras after nuclear reprogramming with exogenous factors. The Journal of Biological Chemistry.

[17] Wang L, Wang L, Huang W, Su H, Xue Y, Su Z, Liao B, Wang H, Bao X, Qin D, He J, Wu W, So KF, Pan G, Pei D (2013). Generation of integration-free neural progenitor cells from cells in human urine. Nature Methods.

[18] Lian X, Bao X, Zilberter M, Westman M, Fisahn A, Hsiao C, Hazeltine LB, Dunn KK, Kamp TJ, Palecek SP (2015). Chemically defined, albumin-free human cardiomyocyte generation. Nature Methods.

[19] Buenrostro JD, Giresi PG, Zaba LC, Chang HY, Greenleaf WJ (2013). Transposition of native chromatin for fast and sensitive epigenomic profiling of open chromatin, DNA-binding proteins and nucleosome position. Nature Methods.

[20] Buenrostro, J. D., Wu, B., Chang, H. Y., & Greenleaf, W. J. (2015). ATAC-seq: A method for assaying chromatin accessibility genome-wide. *Curr Protoc Mol biol, 109, *21 29 21–21 29 29*. *10.1002/0471142727.mb2129s109 PMC4374986 25559105

[21] Hutchins AP, Yang Z, Li Y, He F, Fu X, Wang X, Li D, Liu K, He J, Wang Y, Chen J, Esteban MA, Pei D (2017). Models of global gene expression define major domains of cell type and tissue identity. Nucleic Acids Research.

[22] Li, B., & Dewey, C. N. (2011). RSEM: Accurate transcript quantification from RNA-Seq data with or without a reference genome. *BMC bioinformatics, 12, *323. 10.1186/1471-2105-12-323 PMC3163565 21816040

[23] Langmead B, Salzberg SL (2012). Fast gapped-read alignment with bowtie 2. Nature Methods.

[24] Risso, D., Schwartz, K., Sherlock, G., & Dudoit, S. (2011). GC-content normalization for RNA-Seq data. *BMC bioinformatics, 12, *480. 10.1186/1471-2105-12-480 PMC3315510 22177264

[25] Young MD, Wakefield MJ, Smyth GK, Oshlack A (2010). Gene ontology analysis for RNA-seq: Accounting for selection bias. Genome Biology.

[26] Ramirez, F., Dundar, F., Diehl, S., Gruning, B. A., & Manke, T. (2014). deepTools: a flexible platform for exploring deep-sequencing data. *Nucleic acids res, 42*(web server issue), W187–191. 10.1093/nar/gku365 PMC4086134 24799436

[27] Heinz S, Benner C, Spann N, Bertolino E, Lin YC, Laslo P, Cheng JX, Murre C, Singh H, Glass CK (2010). Simple combinations of lineage-determining transcription factors prime cis-regulatory elements required for macrophage and B cell identities. Molecular Cell.

[28] Hutchins AP, Jauch R, Dyla M, Miranda-Saavedra D (2014). Glbase: A framework for combining, analyzing and displaying heterogeneous genomic and high-throughput sequencing data. Cell Regen.

[29] Lim LS, Hong FH, Kunarso G, Stanton LW (2010). The pluripotency regulator Zic3 is a direct activator of the Nanog promoter in ESCs. Stem Cells.

[30] Yang SH, Andrabi M, Biss R, Murtuza Baker S, Iqbal M, Sharrocks AD (2019). ZIC3 controls the transition from naive to primed pluripotency. Cell Reports.

[31] Hatayama M, Tomizawa T, Sakai-Kato K, Bouvagnet P, Kose S, Imamoto N, Yokoyama S, Utsunomiya-Tate N, Mikoshiba K, Kigawa T, Aruga J (2008). Functional and structural basis of the nuclear localization signal in the ZIC3 zinc finger domain. Human Molecular Genetics.

[32] Yu FX, Zhao B, Guan KL (2015). Hippo pathway in organ size control, tissue homeostasis, and cancer. Cell.

[33] Zhao B, Ye X, Yu J, Li L, Li W, Li S, Yu J, Lin JD, Wang CY, Chinnaiyan AM, Lai ZC, Guan KL (2008). TEAD mediates YAP-dependent gene induction and growth control. Genes & Development.

[34] Nishioka N, Inoue K, Adachi K, Kiyonari H, Ota M, Ralston A, Yabuta N, Hirahara S, Stephenson RO, Ogonuki N, Makita R, Kurihara H, Morin-Kensicki EM, Nojima H, Rossant J, Nakao K, Niwa H, Sasaki H (2009). The hippo signaling pathway components Lats and yap pattern Tead4 activity to distinguish mouse trophectoderm from inner cell mass. Developmental Cell.

[35] Shi Z, He F, Chen M, Hua L, Wang W, Jiao S, Zhou Z (2017). DNA-binding mechanism of the hippo pathway transcription factor TEAD4. Oncogene.

[36] Tian W, Yu J, Tomchick DR, Pan D, Luo X (2010). Structural and functional analysis of the YAP-binding domain of human TEAD2. Proceedings of the National Academy of Sciences of the United States of America.

[37] Joo JS, Cho SY, Rou WS, Kim JS, Kang SH, Lee ES, Moon HS, Kim SH, Sung JK, Kwon IS, Eun HS, Lee BS (2020). TEAD2 as a novel prognostic factor for hepatocellular carcinoma. Oncology Reports.

[38] Tamm C, Bower N, Anneren C (2011). Regulation of mouse embryonic stem cell self-renewal by a yes-YAP-TEAD2 signaling pathway downstream of LIF. Journal of Cell Science.

[39] Xing QR, El Farran CA, Gautam P, Chuah YS, Warrier T, Toh CXD, Kang NY, Sugii S, Chang YT, Xu J, Collins JJ, Daley GQ, Li H, Zhang LF, Loh YH (2020). Diversification of reprogramming trajectories revealed by parallel single-cell transcriptome and chromatin accessibility sequencing. Science Advances.

[40] Fu Q, Qin Z, Jin X, Zhang L, Chen Z, He J, Ji J, Yao K (2017). Generation of functional lentoid bodies from human induced pluripotent stem cells derived from urinary cells. Investigative Ophthalmology & Visual Science.

[41] Mauritz C, Martens A, Rojas SV, Schnick T, Rathert C, Schecker N, Menke S, Glage S, Zweigerdt R, Haverich A, Martin U, Kutschka I (2011). Induced pluripotent stem cell (iPSC)-derived Flk-1 progenitor cells engraft, differentiate, and improve heart function in a mouse model of acute myocardial infarction. European Heart Journal.

[42] Mulder J, Sharmin S, Chow T, Rodrigues DC, Hildebrandt MR, D'Cruz R, Rogers I, Ellis J, Rosenblum ND (2020). Generation of infant- and pediatric-derived urinary induced pluripotent stem cells competent to form kidney organoids. Pediatric Research.

[43] Song J, Yang X, Zhou Y, Chen L, Zhang X, Liu Z, Niu W, Zhan N, Fan X, Khan AA, Kuang Y, Song L, He G, Li W (2019). Dysregulation of neuron differentiation in an autistic savant with exceptional memory. Molecular Brain.

[44] Wang C, Hei F, Ju Z, Yu J, Yang S, Chen M (2016). Differentiation of urine-derived human induced pluripotent stem cells to alveolar type II epithelial cells. Cellular Reprogramming.

