# Supplementary figures and images for: Identification of New Transcription Factors that Can Promote Pluripotent Reprogramming
Source: Stem Cell Rev Rep. 2021 Aug 26;17(6):2223–34. doi: 10.1007/s12015-021-10220-z (PMC8599342; doi:10.1007/s12015-021-10220-z)

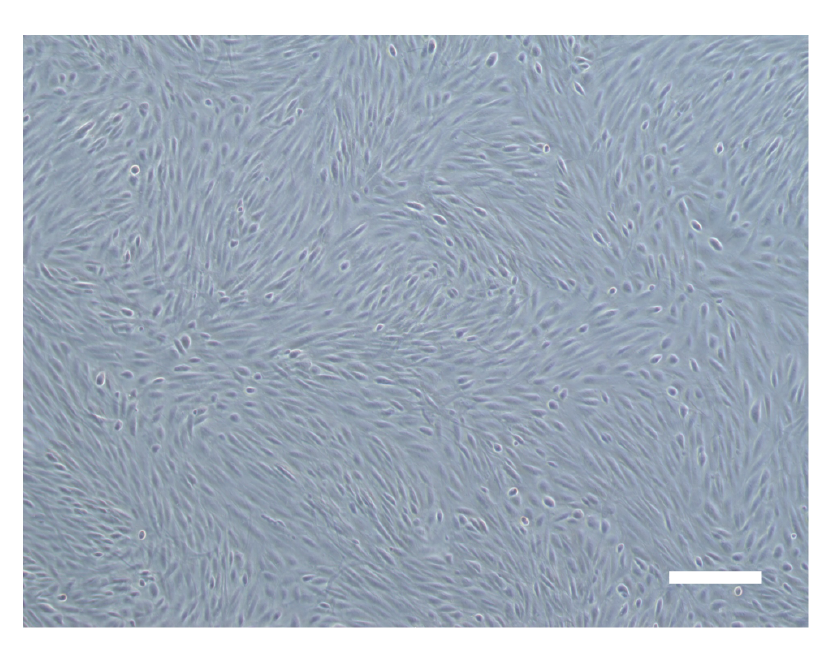

Supplement: Supplementary file 1 — Morphology of urine cells obtained from volunteer donors (before induction) (bar: 200 μm). (PNG 1622 kb) [file 12015_2021_10220_Fig5_ESM.png]

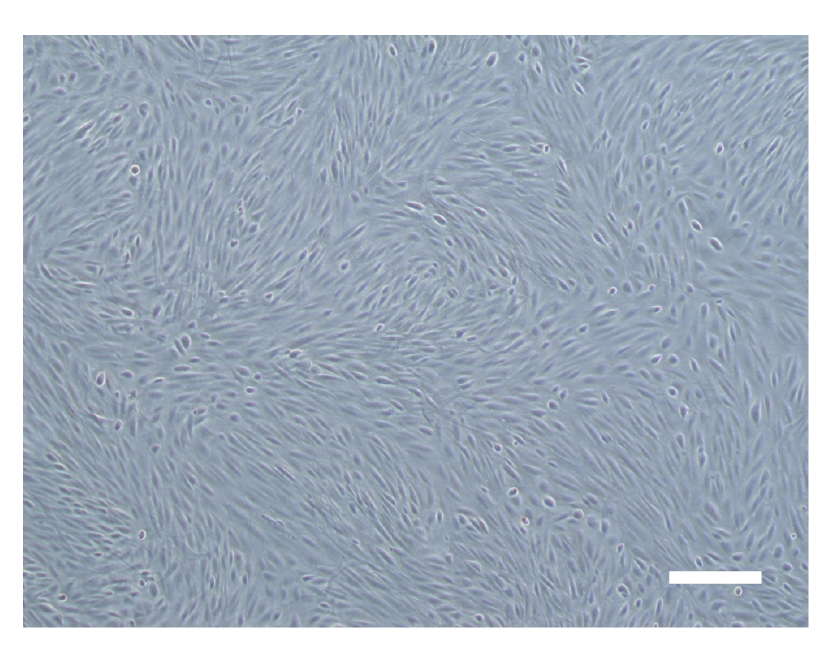

Supplement: Supplementary file 2 — High resolution image (TIF 2346 kb) [file 12015_2021_10220_MOESM1_ESM.tif]

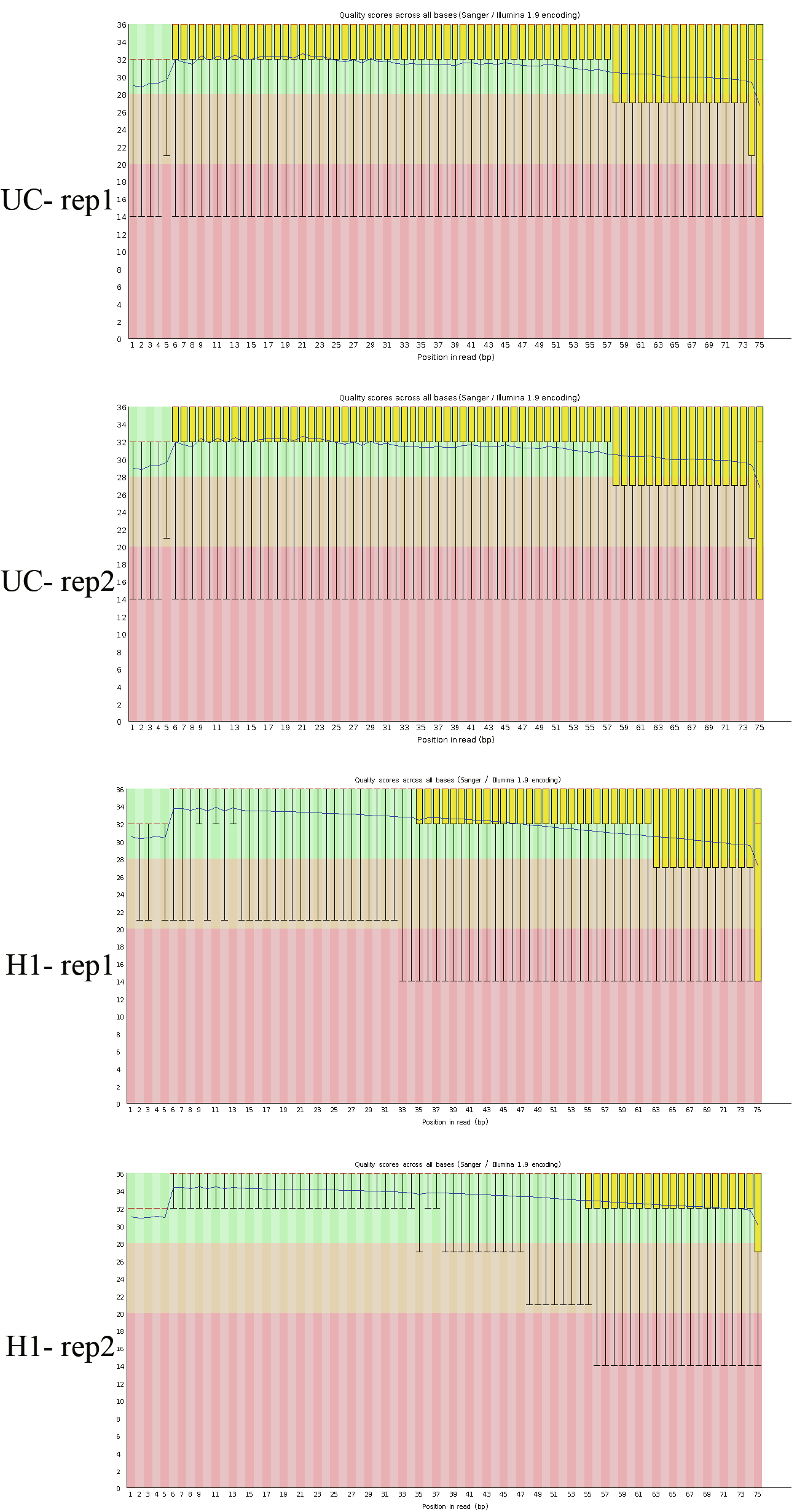

Supplement: Supplementary file 3 — Per base sequence quality of FASTQC Report of ATAC-seq. UC rp1 and UC rp2 represent urine cell samples from two donors, respectively. H1 rp1 and H1 rp2 represent two ATAC-seq libraries of human embryo stem cells. (PNG 9361 kb) [file 12015_2021_10220_Fig6_ESM.png]

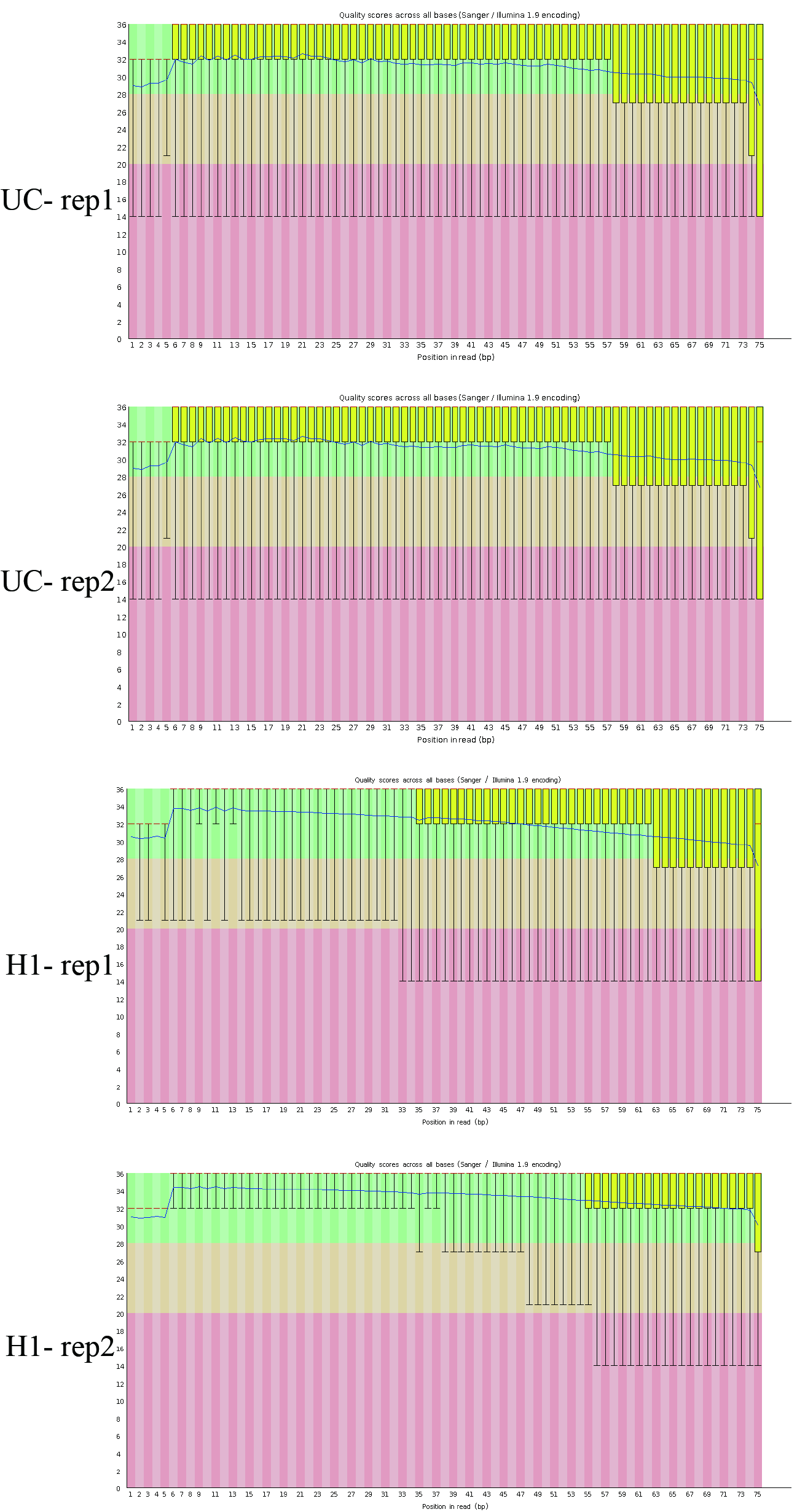

Supplement: Supplementary file 4 — High resolution image (TIF 3005 kb) [file 12015_2021_10220_MOESM2_ESM.tif]

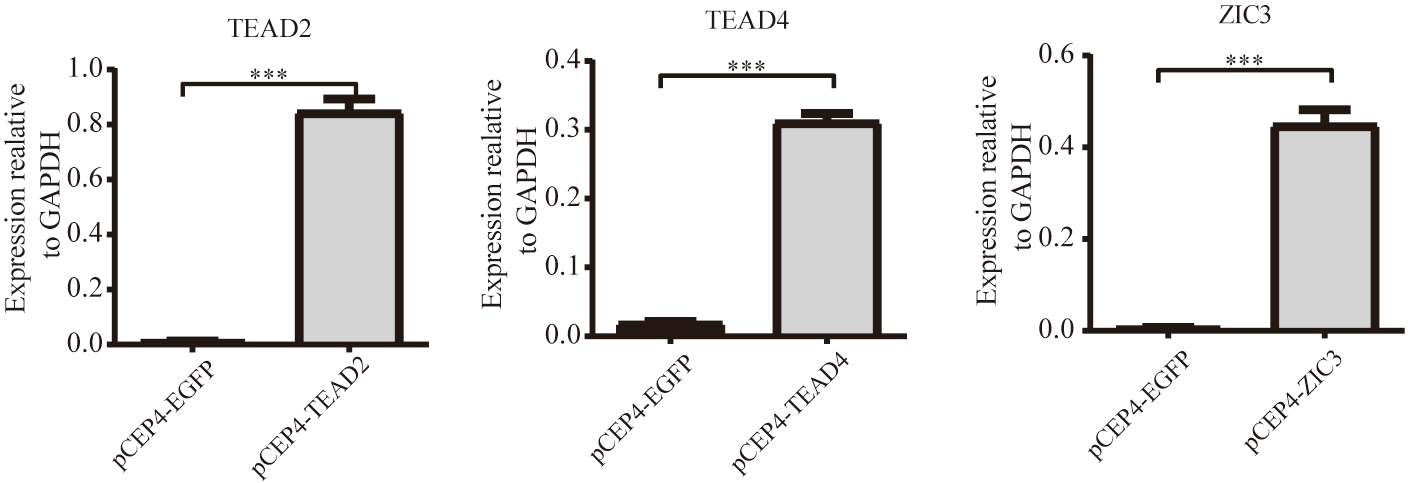

Supplement: Supplementary file 5 — After transiently transfecting pCEP4-TEAD2, pCEP4-TEAD4 and pCEP4-ZIC3 into HEM-293 T cells, the expression of TEAD2, TEAD4 and ZIC3 was significantly higher than control cells transfected with pCEP4-GFP. Data represented as mean ± SEM from three independent assays. *P < 0.05, **P < 0.01, ***P < 0.001. (PNG 1995 kb) [file 12015_2021_10220_Fig7_ESM.png]

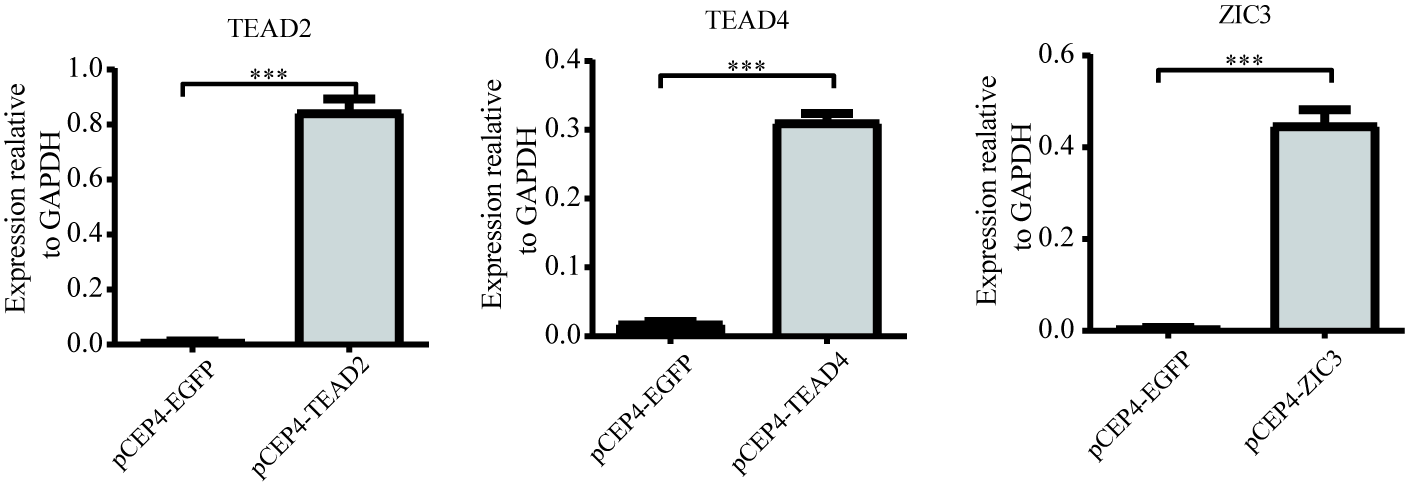

Supplement: Supplementary file 6 — High resolution image (TIF 738 kb) [file 12015_2021_10220_MOESM3_ESM.tif]

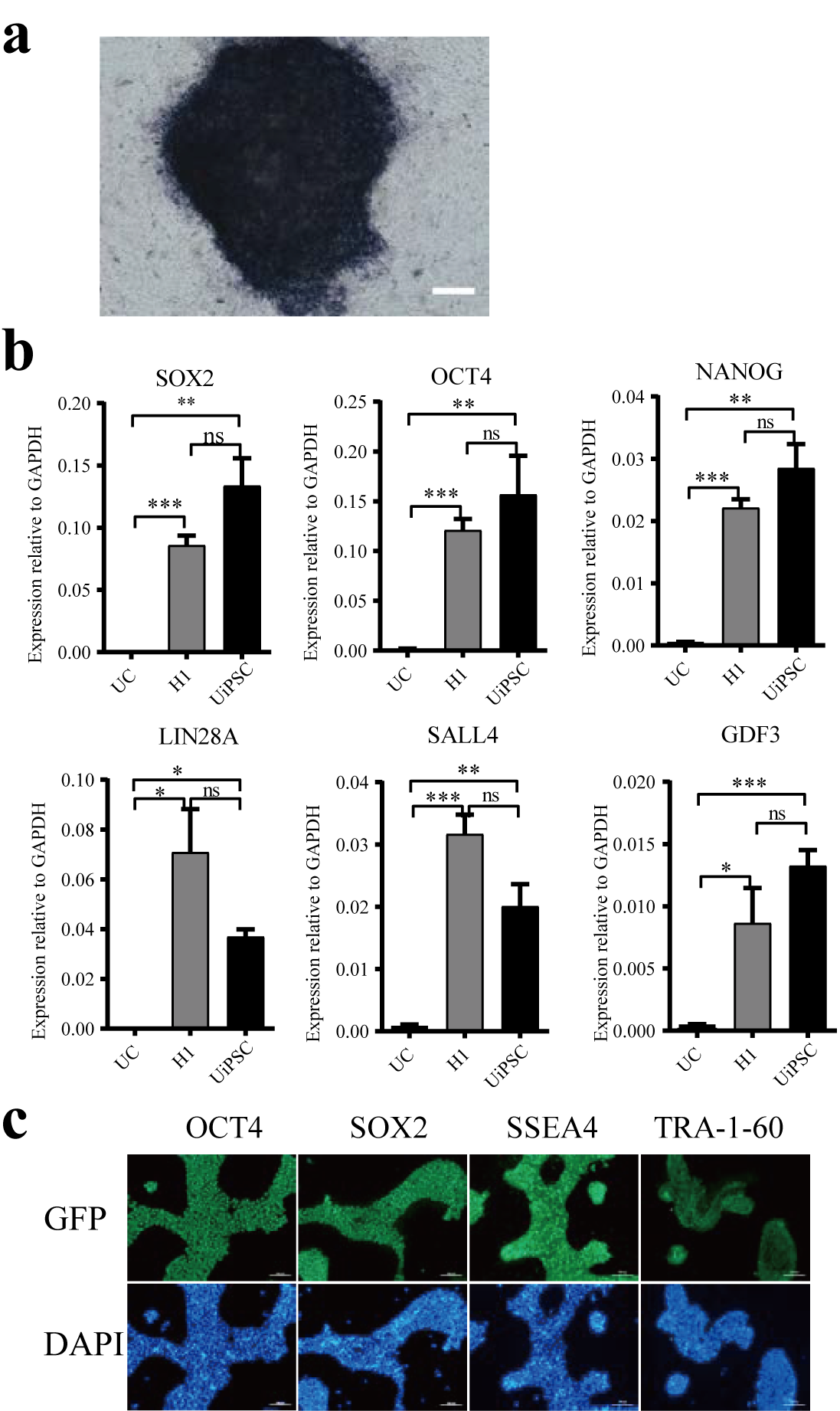

Supplement: Supplementary file 7 — The characteristics of UiPSCs. a AP staining shown AP positive clones after 17 days iPSCs reprogramming with OSK plus miR302–367 cluster (bar = 100 μm). b The expression level of pluripotent genes OCT4, SOX2, NANOG, LIN28A, SALL4, GDF3 in UiPSCs, H1 and UCs. Data represented as mean ± SEM from three independent assays. Data represented as mean ± SEM from three independent assays. *P < 0.05, **P < 0.01, ***P < 0.001. c Immunostaining of OCT4, SOX2, SSA4, TRA-1-60 in UiPSCs (bar: 100 μm). (PNG 5925 kb) [file 12015_2021_10220_Fig8_ESM.png]

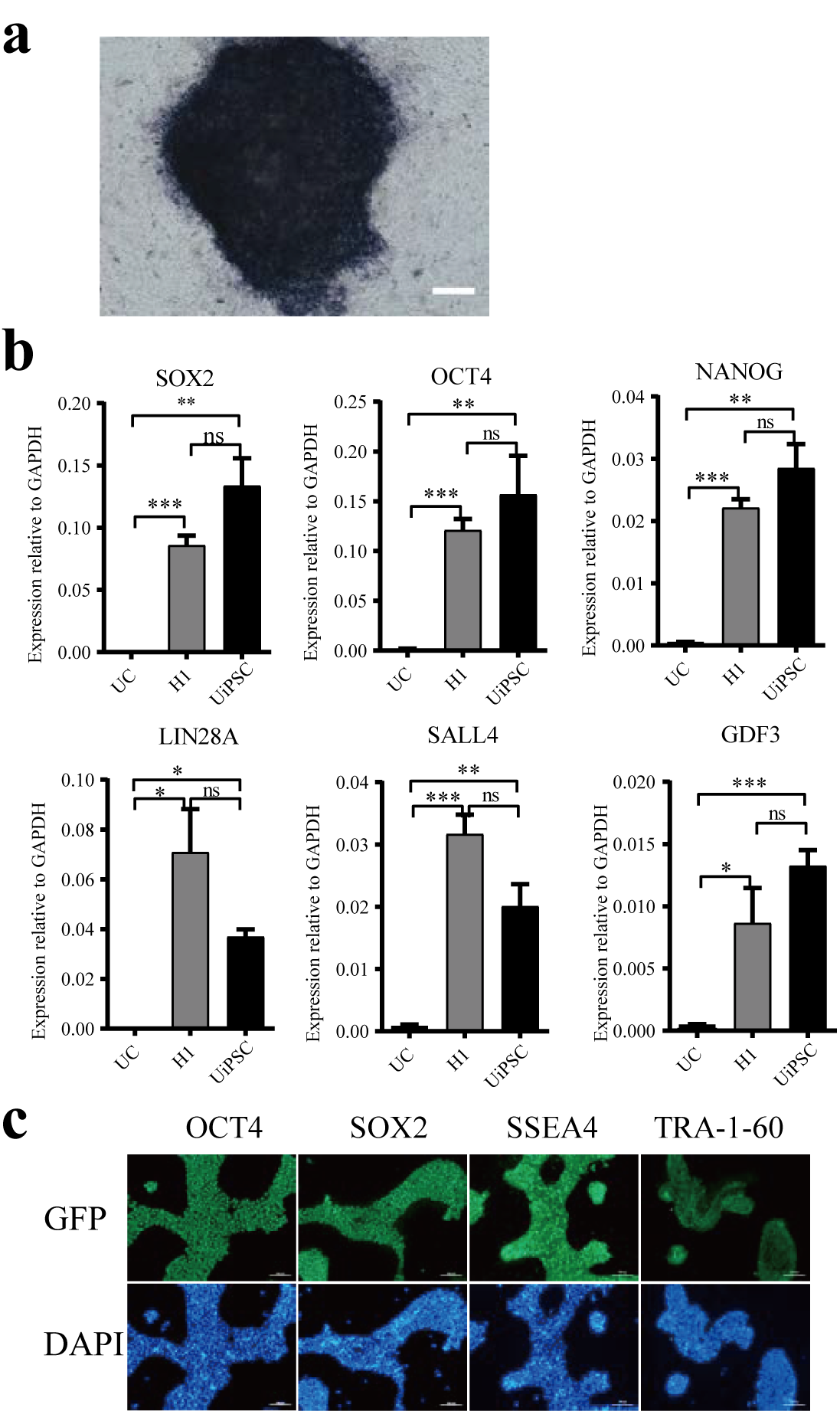

Supplement: Supplementary file 8 — High resolution image (TIF 2291 kb) [file 12015_2021_10220_MOESM4_ESM.tif]
